# Anterior Urethral Stricture Disease Negatively Impacts the Quality of Life of Family Members

**DOI:** 10.1155/2016/3582862

**Published:** 2016-03-01

**Authors:** Jonathan R. Weese, Valary T. Raup, Jairam R. Eswara, Stephen D. Marshall, Andrew J. Chang, Joel Vetter, Steven B. Brandes

**Affiliations:** ^1^Division of Urology, Department of Surgery, Washington University School of Medicine, Saint Louis, MO 63110, USA; ^2^Division of Urology, Department of Surgery, Brigham and Women's Hospital, Harvard Medical School, Boston, MA 02115, USA; ^3^Laconia Clinic Urology, Laconia, NH 03246, USA; ^4^Chesapeake Urology, Baltimore, MD 21061, USA; ^5^Department of Urology, Columbia University Medical Center, New York, NY 10032, USA

## Abstract

*Purpose*. To quantify the quality of life (QoL) distress experienced by immediate family members of patients with urethral stricture via a questionnaire given prior to definitive urethroplasty. The emotional, social, and physical effects of urethral stricture disease on the QoL of family members have not been previously described.* Materials and Methods*. A questionnaire was administered prospectively to an immediate family member of 51 patients undergoing anterior urethroplasty by a single surgeon (SBB). The survey was comprised of twelve questions that addressed the emotional, social, and physical consequences experienced as a result of their loved one.* Results*. Of the 51 surveyed family members, most were female (92.2%), lived in the same household (86.3%), and slept in the same room as the patient (70.6%). Respondents experienced sleep disturbances (56.9%) and diminished social lives (43.1%). 82.4% felt stressed by the patient's surgical treatment, and 83.9% (26/31) felt that their intimacy was negatively impacted.* Conclusions*. Urethral stricture disease has a significant impact on the family members of those affected. These effects may last decades and include sleep disturbance, decreased social interactions, emotional stress, and impaired sexual intimacy. Treatment of urethral stricture disease should attempt to mitigate the impact of the disease on family members as well as the patient.

## 1. Introduction

It is well known that urethral stricture disease can lead to poor quality of life (QoL) for patients [1–4]. Strictures cause obstructive voiding symptoms, often requiring numerous urethral dilations, direct visual internal urethrotomies (DVIUs), or urethroplasties to achieve definitive resolution. Many patients experience chronic infections and urethral pain, although a minority of patients may develop more severe complications of acute urinary retention, detrusor myogenic failure, urethrocutaneous fistulae, renal failure, or sepsis [3–5]. It has been reported that up to 44% of patients experience sexual dysfunction, particularly in cases of urethral stricture secondary to failed hypospadias repair or lichen sclerosis [6, 7].

Despite the volume of literature discussing the QoL of urethral stricture patients, the effects of this disease on the emotional, social, and physical health of family members have not been previously described. Here, we attempt to quantify the QoL distress experienced by the immediate family members of patients being treated for urethral stricture disease.

## 2. Methods

A unique questionnaire was created to assess the QoL of family members of urethral stricture disease patients (Appendix). This questionnaire was completed by an immediate family member of 51 patients undergoing anterior urethroplasty by a single surgeon from 2013 to 2014 after obtaining Institutional Review Board (IRB) approval. Surveys were administered to the person who accompanied the patient to the hospital on the operative day, which in most cases was the patient's spouse or an immediate family member. The questionnaire was designed to assess the emotional, social, and physical consequences experienced by a close family member as a direct result of their loved one and was administered preoperatively. When designing the questionnaire, relevant urethral stricture disease quality of life issues were determined by qualitative interviews with patients and family members, input from reconstructive urologists, and literature review of typical urethral stricture disease symptomatology. The specific questions were formulated to capture issues that were caused exclusively by the patient's urinary dysfunction. Questions covered the age, sex, living and sleeping arrangements of the loved ones, and how the patient's stricture disease affects the loved one's sleeping habits, social life, ability to complete typical daily tasks, stress level, and, if applicable, sexual intimacy with the patient. Descriptive statistics of categorical variables focused on frequencies and proportions. Medians and ranges were reported for continuously coded variables.

## 3. Results

All patients underwent an anterior urethroplasty, with a mean age of 39.8 years (18–64) and mean stricture length of 5.1 cm (2–14 cm). Stricture development was secondary to hypospadias (2), trauma (7), radiation (1), and idiopathic etiologies (41). The mean number of DVIU/dilations or urethroplasties prior to definitive urethroplasty was 3.9 (0–22) and 0.5 (0–2), respectively. The mean years of voiding dysfunction from urethral stricture was 17 years (0.5–55 yrs). Mean International Prostate Symptom Score (IPSS) was 21 (14–31).

Of the 51 family members, the mean age was 48.9 years (range = 25–83), 92.2% were female (47/51) and 7.8% male (4/51). 86.3% lived in the same household and 70.6% slept in the same room as the patient (44/51, 36/51). 56.9% endorsed sleep disturbances and, of those, 24.1% felt that their sleep was severely disturbed (29/51, 7/29). 43.1% felt that their social life was limited, and of those, 18.2% described this social limitation as “substantial” (22/51, 4/22). However, only 15.7% felt limited by an inability to take care of daily tasks (8/51). 86.3% felt pity for the patient and 82.4% felt stressed by the patient's surgical treatment (44/51, 42/51). 60.8% of respondents were sexually active with the patient, and among these, 83.9% felt that their intimacy was negatively impacted (31/51, 26/31). Of the 83.9% endorsing negative effects on their intimacy, 50% felt that their intimacy was impacted a great deal (13/26).

## 4. Discussion

It has been shown time and time again that urethral stricture disease is detrimental to patient QoL [1–4]. Patients frequently experience voiding symptoms such as weak urinary stream, incomplete emptying, splayed urinary stream, and dysuria, as well as resting lower urinary tract pain and sexual dysfunction [6]. Patients may need to void multiple times throughout the night due to the nature of the disease, which could certainly disturb family members in the same bed, or even in the same home. Most family members (56.9%) felt that their sleep habits were negatively affected due to their partner's symptoms, 24.1% of which endorsed severe sleep disturbances. 86.3% felt pity for the patient and 82.4% felt stressed by the patient's surgical treatment.

Whybrow et al. described the elaborate plans and routines developed by stricture patients to hide their disease from others and help cope with the obstructive voiding symptoms caused by their urethral stricture disease. Some of these patients even described an inability to attend normal social functions due to the severity of their symptoms [8]. These social intricacies do not only affect the patient, they also take a toll upon the family members, loved ones, and caretakers who interact with the patient regularly. In our study, 43.1% felt that their social life was limited, and of those, 18.2% described this social limitation as “substantial.”

Data regarding sexual dysfunction within the stricture population is limited, and reported rates have been highly variable. Nuss et al. reported sexual dysfunction within 11% of all stricture disease patients and up to 24% of patients with prior hypospadias or lichen sclerosis [6]. In contrast, Erickson et al. published rates of preoperative erectile dysfunction in 44% of patients, with 25% complaining of ejaculatory dysfunction [7]. In our study, a strong majority of family members in a sexual relationship with the patient reported diminished sexual intimacy (83.9%). Of the respondents endorsing negative effects on their intimacy, 50% felt that their intimacy was impacted a great deal. This rate is higher than the rates of patient sexual dysfunction reported within the literature, suggesting that sexual dysfunction may be even more prevalent when the patients' intimate partner's opinions are considered. Despite having erections adequate for sexual activity, intimacy between partners is a multifactorial entity. Stricture patients often have Foley catheters or suprapubic catheters that may physically impair sexual contact. In addition, the presence of these tubes and any accompanying urine odor may diminish libido. Although difficult to measure objectively, embarrassment and/or decreased self-confidence due to the patient's urologic condition may also impact sexual intimacy. Thus, stricture disease symptomatology and its subsequent psychological effects have the potential to impact sexual function in a plethora of ways [9].

To our knowledge, no prior studies have investigated the effect of stricture disease on family member QoL. However, there are studies investigating the effect of urinary incontinence on caregiver quality of life. Flaherty et al. first reported that caregiver burden was greater when patients suffered from urinary incontinence and then Gotoh et al. used a validated questionnaire to demonstrate this increased psychological burden [10, 11]. It has also been shown that the QoL of family members is an important consideration when managing all diseases, as family member stress and psychological burden may further diminish patient QoL, as well as overall care and recovery [12]. In addition, diminishing QoL of family members could serve as a surrogate marker of disease severity. Sells et al. reported that partner morbidity correlates with disease severity when considering a patient population with lower urinary tract symptoms caused by benign prostatic hyperplasia [13]. These studies suggest that the treatment plan should include strategies to mitigate the distress felt by family members, such as family/spousal conferences and therapy referrals. Realistic expectations must be established early in the course of disease, and healthy coping mechanisms for the patient and family members should be encouraged.

Our study is not without limitations. A single surgeon (SBB) performed all urethroplasties and all surveys were administered at a single institution, both of which may limit generalizability. Additionally, individual QoL is inherently multifactorial, and the specific issues investigated by our questionnaire are influenced by a number of factors other than the patient's stricture disease. A validated questionnaire would increase the strength of our conclusions. Jackson et al. created a validated patient-reported outcome measure to quantify the effects of urethral stricture surgery, but a validated questionnaire to assess QoL effects on family members and caretakers does not yet exist [14, 15]. Further directions of study include administration of this survey to more patients and at other institutions, as well as at multiple time points along the urethral stricture disease algorithm, including following successful urethroplasty, in order to evaluate how disease progression and treatment affect the QoL of patients and family members.

## 5. Conclusions

Urethral stricture disease has a significant impact on family members and caregivers, particularly spouses and partners. The effects on family members may last decades and include sleep disturbance, decreased social interactions, emotional stress, and impaired sexual intimacy. The elements of an effective treatment strategy, including the timing of definitive urethroplasty, are multifactorial and highly patient-specific. While the QoL of the patient is an extremely important consideration, the QoL of immediate family members must not be ignored. Thus, treatment of urethral stricture disease should include family conferences focusing on expectations, basic stricture education, treatment options, and access to counseling services. Further studies may help to elucidate the ideal management strategy to optimize both patient and family quality of life.

## Appendix

### Stricture Disease: Family Member Survey

1. Do you live in the same home as the patient?
  □ No
  □ Yes
  □ choose not to answer
2. Do you sleep in the same room as the patient?
  □ No
  □ Yes
  □ choose not to answer
3. How are you related to the patient?
  □ Spouse
  □ Partner
  □ friend
  □ family member/Other: —
  □ choose not to answer
4. Your age? —
5. Male or Female (circle one)
6. Do you experience any sleeping disturbances because of the patient's urinary problem?
  □ Not at all
  □ Somewhat
  □ A great deal
  □ choose not to answer
7. Is your social life limited because of the patient's urinary problem
  □ Not at all
  □ Somewhat
  □ A great deal
  □ choose not to answer
8. Is your ability to take care of tasks inside and outside the home affected by the patient's urinary problem?
  □ Not at all
  □ Somewhat
  □ A great deal
  □ choose not to answer
9. Does the patient's urinary problem make you feel pity or sad for them?
  □ Not at all
  □ Somewhat
  □ A great deal
  □ choose not to answer
10. Is the surgical treatment of the patient's condition stressful for you?
  □ Not at all
  □ Somewhat
  □ A great deal
  □ choose not to answer
11. Are you sexually intimate with the patient? If yes, then answer question (12). If no, STOP.
  □ No
  □ Yes
  □ choose not to answer
  □ Not Applicable
12. Is your sexual intimacy affected by the patient's urinary condition?
  □ Not at all
  □ Somewhat
  □ A great deal
  □ choose not to answer.

## Abbreviations

QoL: Quality of life
DVIU: Direct visual internal urethrotomy.
